# Designing a sampling scheme to reveal correlations between weeds and soil properties at multiple spatial scales

**DOI:** 10.1111/wre.12184

**Published:** 2015-11-20

**Authors:** H Metcalfe, A E Milne, R Webster, R M Lark, A J Murdoch, J Storkey

**Affiliations:** ^1^ Rothamsted ResearchHarpendenHertfordshireUK; ^2^ School of AgriculturePolicy and DevelopmentUniversity of ReadingEarley GateReadingUK; ^3^ British Geological SurveyKeyworthNottinghamUK; ^4^ MontanaUSA

**Keywords:** weed patches, nested sampling, reml, geostatistics, black‐grass, *Alopecurus myosuroides*, soil

## Abstract

Weeds tend to aggregate in patches within fields, and there is evidence that this is partly owing to variation in soil properties. Because the processes driving soil heterogeneity operate at various scales, the strength of the relations between soil properties and weed density would also be expected to be scale‐dependent. Quantifying these effects of scale on weed patch dynamics is essential to guide the design of discrete sampling protocols for mapping weed distribution. We developed a general method that uses novel within‐field nested sampling and residual maximum‐likelihood (reml) estimation to explore scale‐dependent relations between weeds and soil properties. We validated the method using a case study of *Alopecurus myosuroides* in winter wheat. Using reml, we partitioned the variance and covariance into scale‐specific components and estimated the correlations between the weed counts and soil properties at each scale. We used variograms to quantify the spatial structure in the data and to map variables by kriging. Our methodology successfully captured the effect of scale on a number of edaphic drivers of weed patchiness. The overall Pearson correlations between *A. myosuroides* and soil organic matter and clay content were weak and masked the stronger correlations at >50 m. Knowing how the variance was partitioned across the spatial scales, we optimised the sampling design to focus sampling effort at those scales that contributed most to the total variance. The methods have the potential to guide patch spraying of weeds by identifying areas of the field that are vulnerable to weed establishment.

## Introduction

Many weed species have patchy distributions in arable fields that can be strongly affected by their environments, in particular the soil (Radosevich *et al*., 2007). The spatial variation in soil results from numerous processes operating at several spatial scales, so the variation in some soil properties can also be patchy though not necessarily on the same scales as the weeds. As a consequence, the relations between the abundances of weeds and particular soil properties can change from one spatial scale to another. This means that relations between the two variables found at the one scale might not hold at another (Corstanje *et al*., 2007). In these circumstances, a small absolute correlation coefficient between a weed count and a soil property calculated from a simple random sample over a whole field, although statistically sound, could obscure strong relations at particular scales and be misleading.

Several investigators (e.g. Gaston *et al*., 2001; Walter *et al*., 2002; Nordmeyer & Häusler, 2004) have used grids for studying spatial variation in weeds. They have assumed some prior knowledge of the spatial scales of variation in the field and that has led them to choose grid intervals that would capture the necessary spatial detail; they would not have wished to risk missing such detail by having too coarse a grid. However, sampling at fine scales would make sampling the whole of a large field very expensive and, almost certainly, unnecessarily so if the aim is to understand the general position of patches within the field rather than small changes in the location of patches. These difficulties associated with the design of discrete sampling protocols for studying weed patches, as either a tool for understanding weed ecology or mapping weeds to guide patch spraying, have been thoroughly reviewed by Rew and Cousens (2001). They highlighted the need to develop new analytical techniques to capture the effects of scale on the dynamics of weed patches and to optimise sampling. Partly because of the risk of discrete sampling at too coarse a resolution, they argued that ground‐based continuous sampling was more appropriate for practical site‐specific weed management applications. Whilst many mapping procedures can be carried out early in the season and used for control in the current season, real‐time detection and control is difficult. For many grass weeds, the current systems can only definitively identify the species of grass once it is flowering. This will be too late for the application of selective herbicides (Murdoch *et al*., 2010). It is therefore also necessary to consider the risk of seedlings establishing outside the mapped patch when planning site‐specific herbicide sprays in the following season. An understanding of the edaphic drivers of weed patch dynamics and the scales at which they operate is both of theoretical interest to weed ecologists and could allow these ‘weed vulnerable zones’ to be identified based on maps of soil properties. Here, we address these issues by applying sampling methodologies designed in the field of soil science to optimise sampling effort to the study of weed patches and how they may relate to environmental properties at multiple spatial scales.

We used the model system of *Alopecurus myosuroides* Huds. in winter wheat (*Triticum aestivum* L.) to demonstrate the potential of these methods. The distribution of *A. myosuroides* is patchy and its density seems to depend to some degree on the nature of the soil (Holm, 1997; Lutman *et al*., 2002). We assumed no prior knowledge of the spatial scale(s) on which the weed varied in fields and so we explored its distribution in one particular field by sampling with a nested design followed by a hierarchical statistical analysis to partition the variance and covariances with soil properties according to spatial scale. In principle, nested sampling schemes allow the estimation of the components of variance for a variable across a wide range of spatial scales and to quantify the covariation and correlation between variables over that range. As we did not know beforehand what sizes of patches to expect or whether to expect variation and causal relations with the soil at more than one spatial scale, we designed a nested sampling scheme with a wide range of sampling intervals that we hoped would reveal the spatial scale(s) of variation in the weed and of its covariation with the soil. We used the method proposed by Lark (2011) to optimise our sampling scheme. The aim of the optimisation was to partition the sampling across the scales, so that the estimation errors for the components of variance were as small as possible with the resources available.

Our primary objective was to develop and validate a generic method to examine the relations between weed distributions and environmental properties at multiple spatial scales. We wanted to demonstrate a way of identifying the relevant scale at which the processes affecting weed patch dynamics operate. This could be a precursor to the use of data on environmental heterogeneity to support patch spraying or to guide the design of optimal sampling strategies for studying weed spatial dynamics. The case study reported here demonstrates the use of this methodology in one field and provides evidence to support the hypothesis that relations between soil variables and weed patches are scale‐dependent.

## Materials and methods

### Study site

The field we chose for study is on a commercial farm in Harpenden, Hertfordshire, UK. It has long been in arable cultivation and is infested with *A. myosuroides*. It comprises two former fields from which the old boundary was removed some decades ago. The southern part of the field is generally flat, whilst the northern part slopes gently downwards towards the north. The soil is stony clay loam containing numerous flints and overlies the clay‐with‐flints formation. The soil grades from Batcombe series in the southern part to the somewhat more clay‐rich Winchester series on the northern slope (Hodge *et al*., 1984).

### Sampling scheme

To consider how the *A. myosuroides* patches vary in space and how that variation relates to soil properties at multiple spatial scales, we examined the spatial components of variance and covariance. This allows us to express the patchiness of the weed's distribution in the field statistically. Estimates of the components of variance can describe the infestation at several scales, and from them, one should be able to design better targeted sampling schemes for future surveys.

Youden and Mehlich (1937) first proposed a nested sampling design to discover the spatial scales of variation in soil. They sampled the soil at locations that were organised hierarchically into clusters separated by fixed distances. The nested sampling design had several main stations separated across the region. These correspond to the top level of the design (level 1). Within each main station, they selected two substations (level 2), which were separated by a fixed distance (305 m), but with the vector joining the substations oriented on a random bearing. Within each substation at level 2, they selected a further two substations at level 3, this time separated by 30.5 m. The final level of replication within their design, level 4, was with pairs of substations within each level‐3 substation, separated by 3.05 m. Soil samples were collected at each of the eight level‐4 substations within each main station. An analysis of variance allowed them to partition the variance of each measured soil property into components associated with each level of the nested design.

**Figure 1:**
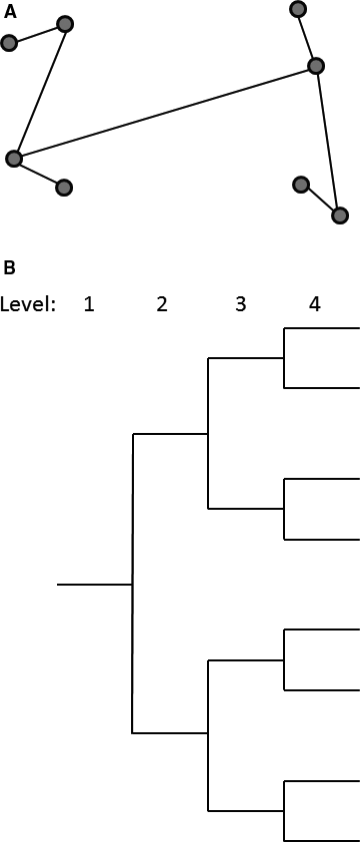
An example of a balanced nested sampling design; (A) the design as it might appear on the ground with circles indicating sampling points, (B) the topological tree from which the design is taken. The design is balanced in that there is equal replication at each level below the first.

This nested design used by Youden and Mehlich (1937) is said to be balanced because any two substations at a given level have identical replication within them at lower levels of the design (Fig. 1). Such designs become prohibitively expensive for more than a few levels, as the number of sample points doubles for every additional level of the design. Furthermore, there are many more fine‐scale comparisons than ones at the coarser scales and this is not necessarily an efficient distribution of sampling effort. For example, in the design shown in Fig. 1, there are four pairs of points separated at the finest scale (level 4), whereas there are only two groups of points separated at level 3 and only one pair of groups of points separated at the coarsest scale within the design, level 2.

Several attempts have been made to economise on nested sampling without seriously sacrificing precision (see Webster *et al*., 2006). Lark (2011) brought together the various strands of that research and proposed designs that are optimal compromises in the sense that they maximise the precision across all levels for given effort, based on the assumption that there is prior knowledge as to how the variation is partitioned across the levels. Here, we apply this approach, for the first time, to the study of weed patches.

The aim of the analysis of a nested sampling design is to estimate components of variance, or covariance, for the sampled variables that correspond to each scale of the hierarchy. As a basis for our study, we adopted the following model:

(1) $$\begin{array}{cc} & z^{u}=x\tau^{u}+\sum_{i=1}^{k}M_{i}\eta_{i}^{u}\\ & z^{v}=x\tau^{v}+\sum_{i=1}^{k}M_{i}\eta_{i}^{v} \end{array}$$

where **z** ^*u*^ comprises *n* random variables by which we model our *n* observations of variable *u* (which is an index, not a power), and similarly for variable *v*, and *k* is the number of random effects in the model. In our case, variable *u* is weed counts and *v* is a measured soil property. One may develop this model for any number of variables. The term **x** *τ* ^*u*^ equates to a vector of mean values for variable *u*. In our case, the mean is constant for any one variable and so comprises the design matrix **x**, which is an *n* × 1 vector of 1s, and *τ* ^*u*^ is the mean for variable *u*. The same applies for variable *v*. The terms in the summation on the right‐hand sides are random effects in the model. There are *k* of these for each variable, each corresponding to one level of the nested sampling scheme, so *k* = 4 in the case shown in Fig. 1. The matrix **M**_*i*_ is a $n\times n_{i}$ design matrix for the *i*th level of the nested scheme, where *n*_*i*_ is the number of sampling stations at the *i*th level across the whole design. If the *m*th sample location belongs to the *m*_*i*_th substation in the *i*th level of the design, then **M**_*i*_[*m*,* m*_*i*_] = 1 and all other elements in the *m*th row are zero. The term $\eta_{i}^{u}$ is an *n*_i_ × 1 random vector. The mean of its elements is zero and their variance is $\sigma_{u,i}^{2}$. This is the variance component for variable *u* associated with the *i*th scale. Similarly, the elements of $\eta_{i}^{v}$ have mean zero and variance $\sigma_{v,i}^{2}$. This multivariate extension of the nested spatial sampling scheme was proposed by Lark (2005) and has been used since in soil science (e.g. Corstanje *et al*., 2007).

One novel aspect of our study was that at the outset, we did not know the spatial scale(s) on which *A. myosuroides* varied, nor whether the variances differed substantially from scale to scale. We therefore assumed the variances to be equal at all scales and designed a sampling scheme accordingly. Our design is as follows, with five levels in the hierarchy.

**Figure 2:**
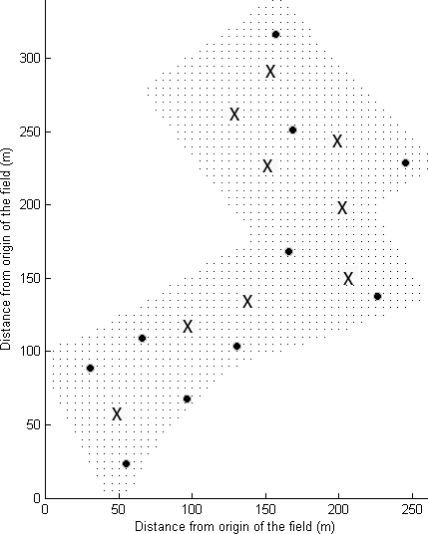
Location of sampling points within the field. The field is marked by grey dots. The locations of the nine main stations are shown as crosses. The 10 extra sampling points are shown as closed discs.

**Figure 3:**
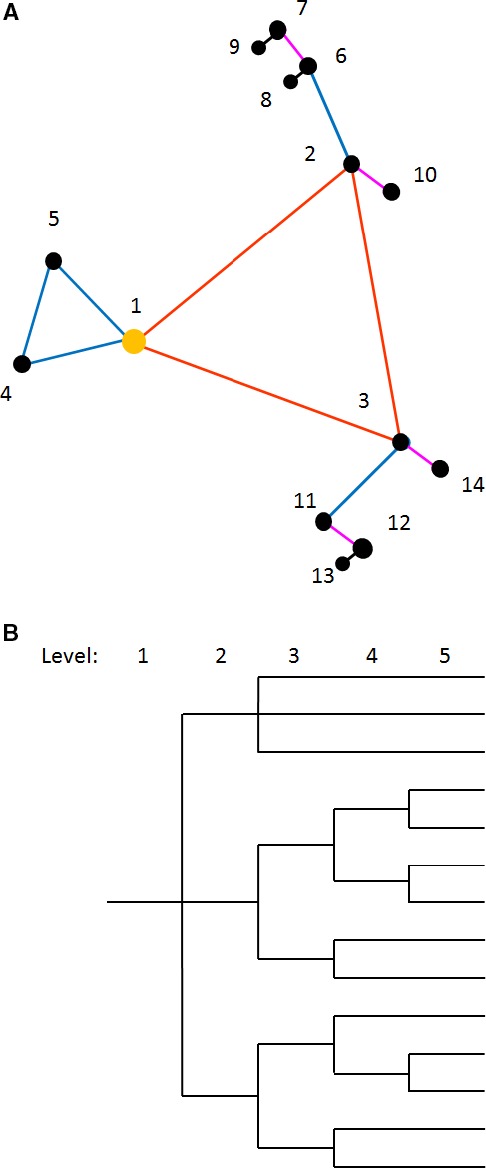
Nested sampling design used in case study (A) the design as one instance might appear on the ground with vertices labelled as the numbers 1–14. The yellow disc indicates the main station of the motif. Red lines represent nodes spaced 20 m apart, blue lines indicate 7.3 m, purple lines link points 2.7 m apart and black lines link those 1 m apart. (B) Topological tree of nested sampling design used in case study. The design is unbalanced as replication is not equal at all branches of the tree.

Nine main stations were spaced approximately 50 m apart across the field (Fig. 2); this corresponds with level 1 of the hierarchy. Sampling sites were nested in groups at each main station (Fig. 3A). The distances between sites at level 2 in the design were 20.0 m, at level 3 the sites were spaced 7.3 m apart, those at level 4 were 2.7 m apart, and those at level 5 were spaced 1.0 m apart. The distances were fixed, but the directional bearings were randomised independently to satisfy the requirements of the model (Eqn (2)). Figure 3B shows the structure as a topological tree, which is evidently unbalanced in that the replication is not equal in all branches of the tree. To improve our maps of *A. myosuroides* distribution and associated soil properties, we added 10 more sampling points, to give a total of 136 sampling points across the field. These additional points were added to fill the larger gaps in the coverage and thereby enable us to diminish the errors in maps made by kriging (Fig. 2).

The positions for the main stations at the 1st level of the design were located in the field by GPS, with subsidiary points located by their distance and orientation from the main station by tape measure and compass. Square quadrats (0.5 m^2^) were placed on the ground with their south‐west vertices at the sampling point. All locations were subsequently geo‐referenced with an RTK GPS (Topcon Positioning Systems, Livermore, CA, USA) with a quoted resolution of 5 cm.

*Alopecurus myosuroides* individuals within each quadrat were counted in late October 2013, while the plants were at the one‐ to two‐leaf stage. No pre‐emergence herbicide had been used on the field.

### Soil analyses

Two cores of soil were taken from each quadrat with a half‐cylindrical auger of diameter 3 cm to a depth of 28 cm on 21 January 2014, while the soil was at field capacity. The depth at which the clay layer was first visible was noted in each of the two augers to indicate the depth of cultivation. If the clay layer was not reached within the 28 cm, then a value of 30 cm was assigned. The average of the two replicates was then recorded. The gravimetric water content was measured in layers 0–10 cm and 10–28 cm by loss on oven‐drying at 105°C. Other variables were measured on samples pooled from the two cores within each quadrat. Organic matter was measured by loss on ignition. Available phosphorus (P) was measured in a sodium bicarbonate extract at pH 8.2. The pH was measured in water, and soil texture (particle‐size distribution) was determined by laser diffraction. Stone content by both volume and mass was measured on a core of 76 mm diameter taken to depth 97 mm from the south‐west outside corner of each quadrat.

### Statistical analyses

A balanced design would lead to a straight‐forward analysis of variance (anova) from which the components of variance are readily estimated. Analysing data from an unbalanced design is more complex. Gower (1962) provided formulae for computing the components from an anova. The method now favoured on theoretical grounds is the residual maximum‐likelihood (reml) estimator due to Patterson and Thompson (1971) and is the one we used. Within the reml model (Eqn (2)), the terms $\eta_{i}^{u}$ and $\eta_{i}^{v}$, *i *=* *1, 2,…., *k* are the random effects. The assumption is that the concatenated 2*n* × 1 random vector [[Z^*u*^]^T^[Z^v^]^T^]^T^ has a joint multivariate normal distribution with 2*n* × 2*n* covariance matrix:

(2) $$V=\sum_{i=1}^{k}\left[\begin{array}{cc} \sigma_{u,i}^{2}M_{i}M_{i}^{T}, & C_{i}^{u,v}M_{i}M_{i}^{T}\\ C_{i}^{u,v}M_{i}M_{i}^{T}, & \sigma_{v,i}^{2}M_{i}M_{i}^{T} \end{array}\right],$$

where the superscript T denotes the transpose of a matrix. The variance and covariance components for each scale are the random effects parameters which are estimated by reml.

We calculated Pearson's correlation coefficients for all data to show correlations when scale is ignored. Note, however, that this does not give an unbiased estimate of the correlation, because it ignores the dependency structure imposed by the sampling and is therefore a somewhat arbitrarily weighted combination of the correlations at different scales. Following partitioning of the components of variance at the different spatial scales, estimates of the correlations ($\hat{\rho }$) at each scale (*i*) between *A. myosuroides* and the soil properties were calculated as:

(3) $${{\hat{\rho }}_{i}}^{u,v}=\frac{{{\hat{C}}_{i}}^{u,v}}{{\hat{\sigma }}_{u,i}{\hat{\sigma }}_{v,i}}$$

where the variables *u* and *v* are *A. myosuroides* counts and the soil property, respectively, and the terms with the hats are the reml estimates of their covariances (*C*) and standard deviations (σ). Where the estimated components of variance given by reml were non‐positive, no associated correlation coefficient was calculated. Confidence intervals for the correlations were calculated by Fisher's *z*‐transform, with degrees of freedom appropriate to the number of sampled pairs at the corresponding level of the design.

Variograms were estimated and modelled from all data points from both the sampling design and the 10 additional points to quantify the spatial structure in the variance of the measured variables. We did this using GenStat (Payne, 2013). Semivariances were calculated by the method of moments (Webster & Oliver, 2007):

(4) $$\hat{\gamma }\left(h\right)=\frac{1}{2m(h)}\sum_{j=1}^{m(h)}{\left\{z\left(x_{j}\right)-z\left(x_{j}+h\right)\right\}}^{2}$$

where *z*(**x**_*j*_) and *z*(**x**_*j*_ **+ h**) are the observed values at two locations separated by lag **h**, and *m*(**h**) is the number of pairs of points at that lag. By incrementing **h,** we obtained an ordered set of values to give the experimental variogram, which is a function of the expected mean squared difference between two random variables, *z*(**x**) and *z*(**x + h**) at locations **x** and **x + h**. The variation appeared to be isotropic and so we treated the lag as a scalar in distance only.

In the case of *A. myosuroides* counts, where the distribution was skewed, a log transformation was used before estimation of the variogram. However, the distribution still did not conform to the assumption of normality, and so we used the method of Cressie and Hawkins (1980) for a more robust estimation of the variogram for this type of data. The computing formula is a modified version of Eqn (5):

(5) $$\hat{\gamma }\left(h\right)=\frac{1}{2}\frac{{\left\{\frac{1}{m(h)}\sum_{j=1}^{m(h)}{\left|z\left(x_{j}\right)-z\left(x_{j}+h\right)\right|}^{\frac{1}{2}}\right\}}^{4}}{0.457+\frac{0.494}{m(h)}+\frac{0.045}{m^{2}\left(h\right)}}$$

Where trend was present in the data, as it was for silt content, we incorporated it in a mixed model of fixed and random effects in the reml estimation of the variogram (Webster & Oliver, 2007).

We mapped the variables across the field by ordinary kriging at points on a 1‐m grid and then contoured the predictions in ArcMap (ESRI). For the variables in which we identified trend and used reml to obtain the variogram, we used universal kriging to take the trend into account.

## Results

Individuals of *A. myosuroides* were found in 95% of the 0.5‐m^2^ quadrats. In total, 3917 *A. myosuroides* seedlings were counted with a mean density of 28.8 per quadrat (Table 1). However, the spatial distribution of *A. myosuroides* plants varied throughout the field and had a strongly skewed distribution. A model was fitted to try and normalise the data. The best fit was obtained for logarithms of the data with an offset of 0.6 added before logging. This removed the skew from the data, but revealed a bimodal distribution. When the field was divided into two at the site of the old field boundary, both populations then fitted a negative binomial distribution, a distribution associated with aggregated populations (Gonzalez‐Andujar & Saavedra, 2003). The soil properties measured were all approximately normal in distribution.

**Table 1:** Summary statistics of species counts and environmental variables

Variate	Mean	Minimum	Maximum	Standard deviation	Skew
*Alopecurus myosuroides* (individuals per quadrat)	28.80	0	326	51.0	3.02
Cultivation depth (cm)	24.90	17.1	30.0	2.74	0.13
Gravimetric water content in top 10 cm (%)	25.63	21.8	30.0	1.86	0.58
Gravimetric water content 10–28 cm depth (%)	23.83	19.3	31.0	2.19	0.55
Organic matter (% wet weight)	4.53	3.0	6.0	0.65	0.45
Available phosphorus (mg L^−1^)	24.70	11.0	54.4	8.30	1.27
pH	6.90	6.13	7.79	0.28	0.24
Sand (% wet weight)	32.10	17.0	51.0	4.85	0.41
Silt (% wet weight)	39.51	25.0	50.0	4.27	0.08
Clay (% wet weight)	28.39	23.0	39.0	3.00	0.85
Volume of Stones (%)	19.2	4.44	38.9	6.67	0.52
Mass of stones (g)	172.5	20.3	387.0	75.43	0.13

**Figure 4:**
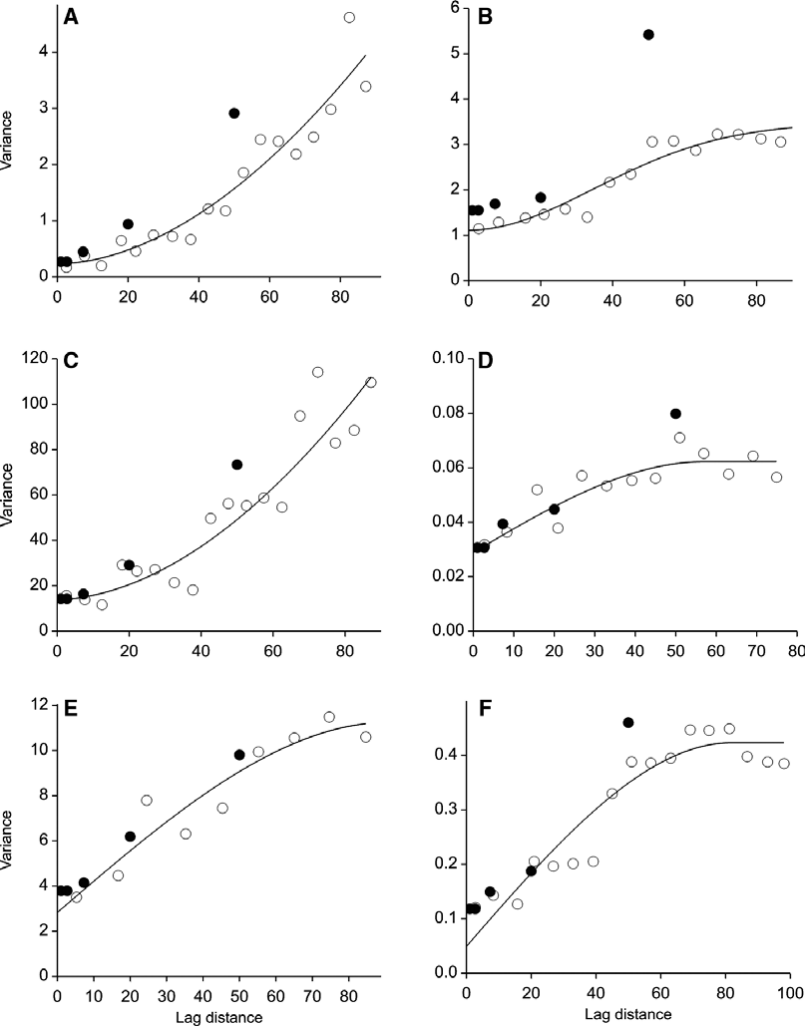
Accumulated components of variance with all negative components of variance set to zero (closed discs) and method of moments variograms (open circles) for (A) *Alopecurus myosuroides*, (B) gravimetric water content in the top 10 cm of soil, (C) available phosphorus, (D) pH, (E) clay content, (F) organic matter. The lags have been binned over all directions and incremented in steps of 6 m. The components of variance plotted at 50 m are calculated from the top level (1) of the design and so encompass all distances >50 m. The solid black lines show the models fitted.

The accumulated components of variance show clear spatial structure in both *A. myosuroides* counts and the soil properties measured (Fig. 4). At fine scales, the variance components estimated by reml analysis were similar to the expected variance obtained from the variogram. However, in most cases the variogram reached a sill at lag distances greater than the maximum distance in the nested design. The functions chosen as models for the variograms were those that best fitted in the least squares sense (Table 2).

**Table 2:** Variogram models fitted to describe the spatial structure in selected measured variables

Variate	Type of model	Nugget	Range	Distance parameter	Sill	Exponent	Linear term
*Alopecurus myosuroides* a	Power	0.229	–	–	–	1.837	0.00101
Gravimetric water content in top 10 cm	Stable^b^	1.110	–	20.23	2.367	–	–
Available phosphorus	Power	13.95	–	–	–	1.837	0.0266
pH	Spherical	0.02890	57.0	–	0.0333	–	–
Clay	Spherical	2.83	91.0	–	8.42	–	–
Organic matter	Spherical	0.0492	82.03	–	0.3742	–	–

a For *Alopecurus myosuroides*, logarithms of the data are used with an offset of 0.6 added before logging.

b The stable model uses an exponent of 0.95.

**Figure 5:**
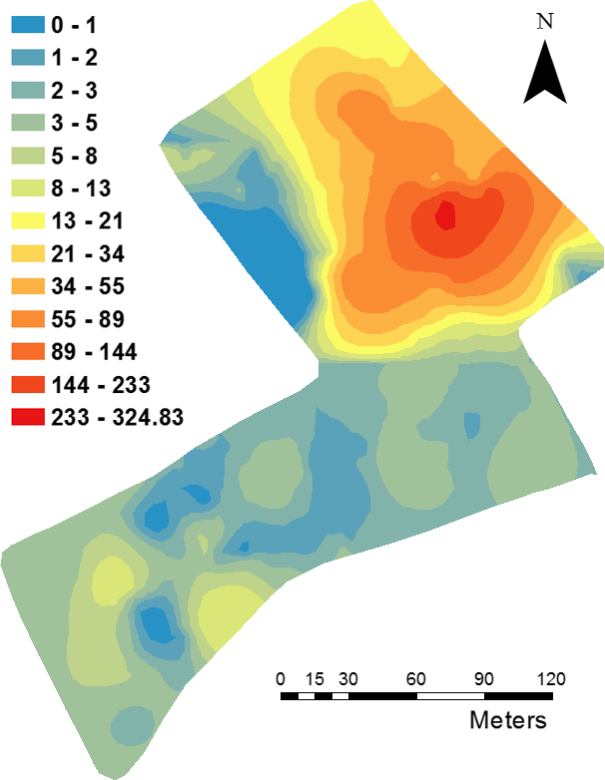
Kriged map of *Alopecurus myosuroides* individuals (per 0.5 m^2^). The model fitted to the experimental variogram of the data is used to provide the best unbiased predictions at points that were not sampled.

**Figure 6:**
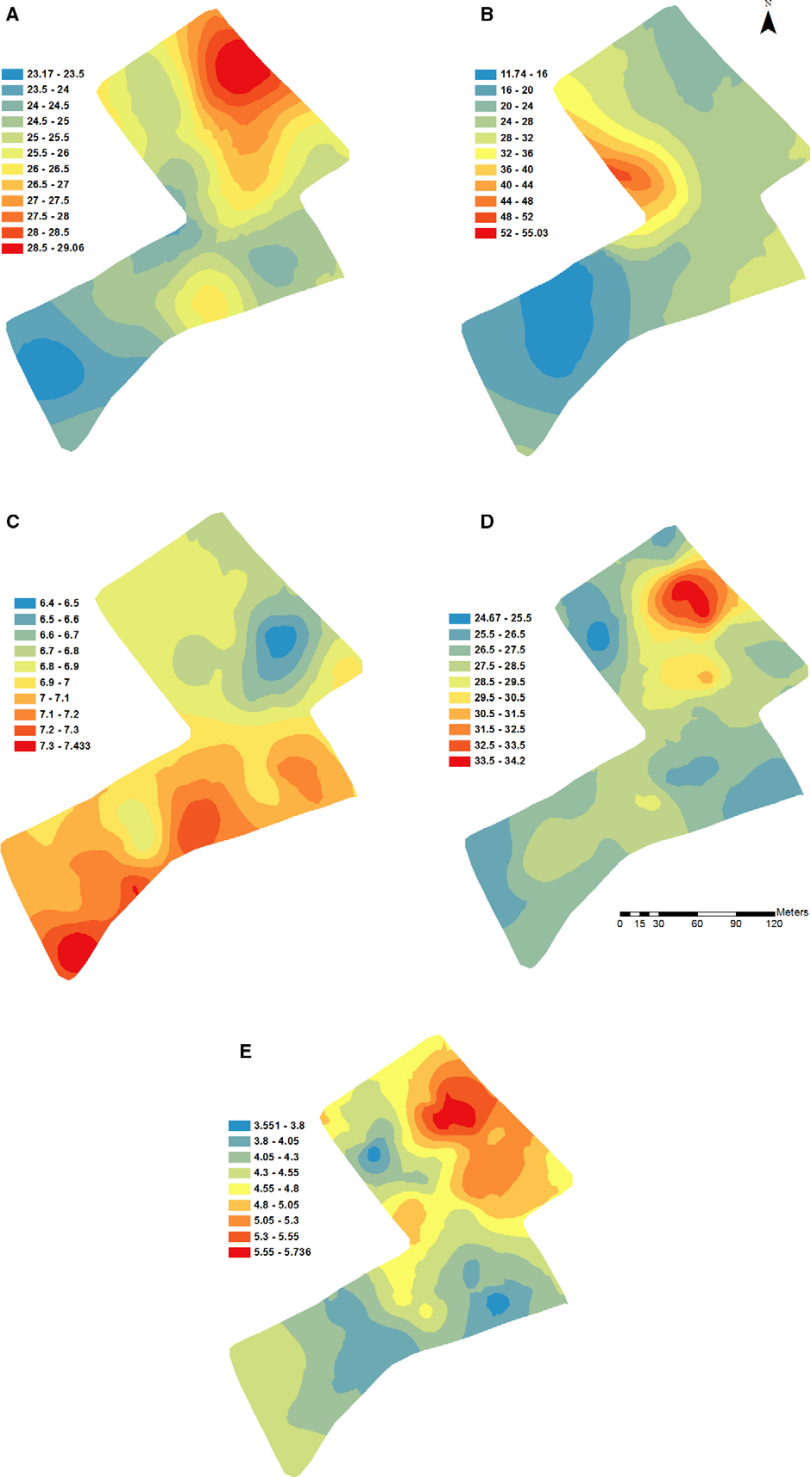
Kriged maps of (A) gravimetric water content in the top 10 cm of soil, (B) available phosphorus (mg L^−1^), (C) pH, (D) clay content and (E) organic matter in soil. In all cases, the models fitted to the experimental variograms of the data are used to provide the best unbiased predictions at unsampled points.

The map of *A. myosuroides* in Fig. 5 was produced by combination of two separate krigings, one for each half of the field, thereby taking into account the bimodal distribution of the weed counts. It shows a large concentration of weeds in the northern part of the field, with only a few seedlings in the southern part of the field. The kriged maps of the soil properties (Fig. 6) show each soil property has a unique spatial distribution. Some of the maps, for example water content (Fig. 6A) and pH (Fig. 6C), show some accord with *A. myosuroides* distribution (Fig. 5).

The statistically significant reml model terms were generally found at the coarsest scales studied here (Table 3), where the covariance terms ($C_{i}^{u,v}$) for each scale (*i* = 1, 2,…, *k*) were set to zero in turn in the reml analysis to test for significance in their contribution to the model.

**Table 3:** Estimated variance components for environmental variables at multiple spatial scales together with the covariance component with *Alopecurus myosuroides* at those scales

Environmental variable	Random term	Estimated variance component for environmental property	Estimated variance component for *A. myosuroides* counts	Estimated covariance component for environmental property and *A. myosuroides*
Gravimetric water content in top 10 cm	lv1	3.603	1.995	2.480*
	lv1.lv2	0.1239	0.4850	0.1401
	lv1.lv2.lv3	0.1484	0.1802	−0.1154
	lv1.lv2.lv3.lv4	−0.2244	−0.00972	0.1387
	Residual variance:			
	lv1.lv2.lv3.lv4.lv5	1.559	0.2620	−0.01321
Available phosphorus	lv1	43.93	1.976	3.150
	lv1.lv2	12.88	0.4960	−1.803*
	lv1.lv2.lv3	2.008	0.1720	0.2699
	lv1.lv2.lv3.lv4	−1.638	−0.01731	−0.1812
	Residual variance:			
	lv1.lv2.lv3.lv4.lv5	13.98	0.2701	0.02844
pH	lv1	0.03577	1.981	−0.2368*
	lv1.lv2	0.005170	0.4940	−0.005534
	lv1.lv2.lv3	0.008005	0.1753	−0.01853
	lv1.lv2.lv3.lv4	−0.004391	−0.02287	−0.01073
	Residual variance:			
	lv1.lv2.lv3.lv4.lv5	0.03132	0.2748	0.02055
Clay	lv1	3.692	1.952	2.294*
	lv1.lv2	1.986	0.4936	0.2752
	lv1.lv2.lv3	0.2887	0.1690	0.1531
	lv1.lv2.lv3.lv4	−0.5752	−0.02259	0.005526
	Residual variance:			
	lv1.lv2.lv3.lv4.lv5	3.904	0.2765	−0.03997
Organic matter	lv1	0.2749	1.963	0.728*
	lv1.lv2	0.03782	0.493	0.00194
	lv1.lv2.lv3	0.02876	0.1725	0.02713
	lv1.lv2.lv3.lv4	−0.01191	−0.01379	0.008752
	Residual variance:			
	lv1.lv2.lv3.lv4.lv5	0.1193	0.2677	−0.00817

Covariances that contributed significantly to the model fitted by reml (*P *<* *0.05) are marked*. Random terms are denoted by lv to signify the level of the hierarchical design, with lv 1 representing the highest level of the design (separate designs across the field) and so corresponds to distances of >50 m and lv2‐5 correspond to distances of 20 m, 7.3 m, 2.7 m and 1 m respectively. All negative estimates for variance components were found not to be statistically significantly different from 0.

**Figure 7:**
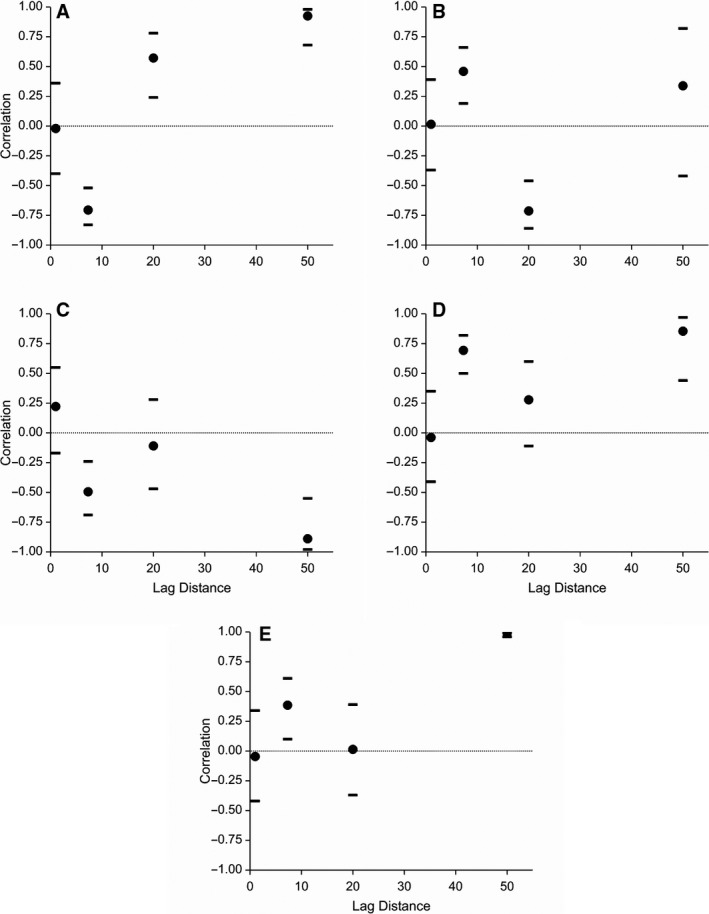
Correlations at the various scales of the nested sampling design between *Alopecurus myosuroides* and (A) water content in the top 10 cm of soil, (B) available phosphorus, (C) pH, (D) clay content and (E) organic matter. Correlations are shown as discs with horizontal bars indicating 95% confidence intervals. The correlations plotted at 50 m are calculated from the top level (1) of the design and so encompass all distances >50 m.

Pearson correlation coefficients between *A. myosuroides* counts and the soil properties were generally weak (Table 4). These take all of the data into account without regard to spatial scale. From these results, we might conclude that there are only weak relations between the density of *A. myosuroides* and the environmental properties measured. However, once the correlations are calculated for the nested design structure, stronger relations are revealed at particular scales (Fig. 7). Often, significant terms in the reml model (Table 3) corresponded with strong correlations between the *A. myosuroides* count and the soil property (Fig. 7), reiterating the likelihood of there being a relation between the weed count and the soil property at that scale.

**Table 4:** Pearson's correlation coefficients between *Alopecurus myosuroides* counts and soil properties measured taking all data into account

Variate	Pearson's correlation coefficient between *A. myosuroides* and the measured variate
Cultivation depth	−0.008
Gravimetric water content in top 10 cm	0.482*
Gravimetric water content 10–28 cm depth	0.491*
Organic matter	0.527*
Available phosphorus	0.023
pH	−0.475*
Sand	0.135
Silt	−0.384*
Clay	0.328*
Volume of stones	0.050
Mass of stones	0.031

Two‐sided tests of correlations different from zero are marked * where significant (*P *<* *0.05).

### Optimising the design

**Figure 8:**
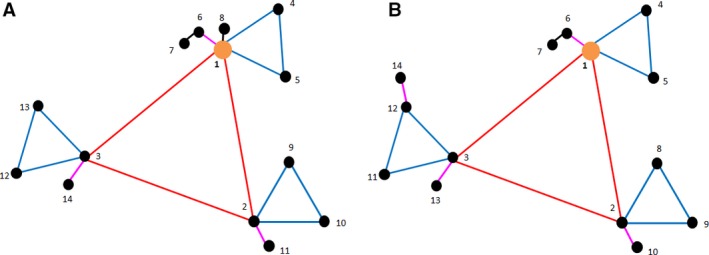
Optimised nested designs with sampling points at vertices (labelled 1—14) as they would appear in the field for (A) the original scales as used in case study (red = 20 m, blue = 7.3 m, purple = 2.7 m, black = 1 m) with optimised topology according to the estimated components of variance from the reml analysis of *Alopecurus myosuroides* counts, (B) the new coarser scales (red = 40 m, blue = 11.5 m, purple = 3.4 m, black = 1 m) with optimised topology according to the estimated components of variance from the model fitted to the variogram of *A. myosuroides* counts.

At the beginning of our study, we had no prior information about the distribution of the variance across scales. Therefore, the nested design we used was based on the assumption of equal variances at all scales. As we now know the components of variance for *A. myosuroides* seedling counts at all scales (Table 5), the sampling design can be optimised as described by Lark (2011). This allows sampling to be focused on the scales that contribute most to the total variance. To achieve this, all components of variance must be positive and so in this example the component of variance for the 4th level is set equal to the minimum positive variance. The optimised design is shown in Fig. 8A.

**Table 5:** Results of REML analysis for log‐transformed *Alopecurus myosuroides* counts. Random terms are denoted by lv to signify the level of the hierarchical design, with lv 1 representing the highest level of the design (separate designs across the field) and so corresponds to distances of >50 m and lv2‐5 correspond to distances of 20 m, 7.3 m, 2.7 m and 1 m respectively

Random term	Estimated variance component	Estimated standard error	Effective degrees of freedom
lv1	1.9759	1.0951	8
lv1.lv2	0.4916	0.2126	18
lv1.lv2.lv3	0.1759	0.0816	34.22
lv1.lv2.lv3.lv4	−0.0176	0.0609	33.19
Residual variance			
lv1.lv2.lv3.lv4.lv5	0.2700	0.0679	31.6

Because of the strong relations observed at the coarse scale between *A. myosuroides* and most of the soil properties, we investigated a wider set of scales increasing exponentially from 1 m at level 5, to 40 m at level 2. This meant the use of distances of 1 m, 3.5 m, 11.5 m and 40 m within the design at each main station. Estimates of the components of variance at each of these distances were taken from the model fitted to the variogram for *A. myosuroides* counts. The component of variance for the top level of the design was set so that the variances had the same sum as the original reml estimates for this field. The design was then optimised for these estimated components of variance. The optimised design at the coarser scales is shown in Fig. 8B.

## Discussion and conclusions

Both the hierarchical analysis and the estimated variogram of the *A. myosuroides* counts revealed clear spatial structure in the data, with observations at short separations showing greater similarity than observations separated by larger distances. Each of the soil variables we measured also had its unique spatial structure that was visible in both the variograms and the components of variance (see Fig. 4). This means that we must recognise the importance of variation at several spatial scales. Within the literature on weed patches, there is a lack of consistency in observed relations with abiotic variables. For example, Walter *et al*. (2002) found a weak negative relation between *Poa annua* L. and organic matter content, whereas Andreasen *et al*. (1991) found a strong positive relation between the two. This lack of consistency may be due to their different sampling scales. Walter *et al*. (2002) sampled on a 20‐m by 20‐m grid, whereas Andreasen *et al*. (1991) randomly selected sample locations within a field. This illustrates the need for more rigorous statistical methods to account for processes operating at different scales.

Despite weak Pearson correlations for all the data (Table 4), covariances and correlations between *A. myosuroides* counts and soil properties showed some strong correlations at various scales. In most instances, the separations that significantly contributed in the reml analyses were the largest of those studied here (>50 m), indicating relations between soil properties and *A. myosuroides* counts occur across the whole field. This is a potentially interesting result in terms of the practical management implications (as we explain below) and warrants further investigation into the scale‐dependent relations between *A. myosuroides* and soil properties. In terms of experimental and analytical methodology, it is particularly important to note how uncorrelated variation between two variables at finer scales can obscure scientifically interesting and practically important relations exhibited at coarser scales, if one were only to examine the overall correlation between variables. The nested sampling scheme and associated analysis set out in this paper are necessary if this problem is to be avoided in experimental studies of the factors affecting weed distribution.

However, other fine‐scale relations not revealed by significant terms in the reml model did appear in the correlations between the weed and soil properties. For example, there were strong positive relations observed at the two coarsest scales between *A. myosuroides* and water content. However, at 7.3 m, there was a negative relation between these two variables, indicating that a different process operates over these smaller distances. So, although *A. myosuroides* establishes most readily in the wettest part of the field, within that wet part establishment was better in the relatively dry parts of it. Similarly for available phosphorus, despite the negligible Pearson correlation between *A. myosuroides* and phosphorus, at 20 m there is a significant negative covariance in the reml model, yet at the 7.3‐m scale, the correlation is positive. This may be explained by depletion of available phosphorus in areas of high weed density (Webster & Oliver, 2007, pp. 220 and 227–228).

We have shown how by nested sampling and hierarchical analysis by reml one can reveal the spatial scale(s) at which weed infestations vary and correlate with soil factors in an economical way. We have also shown how, once one has estimates of components of variance, one can improve a design for future survey without adding substantially to the cost.

These estimates of the components of the variance could be estimated from other more readily available sources of information. For example, the farmer might know something, in a qualitative way, of where and on what spatial scales weeds infest their fields, or the investigator might have access to aerial photography or satellite images that show patchiness in crops or soil that could guide them in designing a sampling scheme. Our methodology is generic and can be used to look at relations between any continuous variable assumed to be related to weed distribution and any weedy variable, whether species distribution or total weed density. We should expect the spatial dependency of soil and weed interactions revealed by the analysis to be context specific. However, ongoing work is seeking to validate the robustness of the relations between soil and *A. myosuroides* patches that emerged from our case study.

This paper has demonstrated how scale‐dependent relations between weed density and soil properties can be examined with appropriate sampling and analysis. The case study showed that such scale‐dependence can occur. It also showed that the nested method may allow us to identify relations that occur at certain scales, but which would be obscured by uncorrelated variations at other scales, if the variables were examined using only the overall correlation for data on a simple random sample. This methodology should be applied to a range of fields with contrasting soil conditions and management strategies, over several seasons, in order to identify scale‐dependent relations between soil and weeds in order to form a basis for a robust strategy for controlling weeds according to the spatial variation in the soil.

Identifying the soil properties that most consistently affect the distribution of *A. myosuroides* in a field could have practical application, if the scale at which the soil and weeds are correlated is appropriate for site‐specific management (as is suggested by our results). Farmers often aim to minimise heterogeneity within individual fields, so that they can treat each field as if it were uniform. Nevertheless, they recognise that there will be some variation within their fields and often have considerable knowledge of that spatial variation (Heijting *et al*., 2011). Now, with modern technology, they can vary their treatment applications accordingly (Lutman *et al*., 2002). Patchy distributions of weeds are particular examples of such heterogeneity. In principle, farmers should be able to control the weeds with herbicide where the weeds occur and avoid using herbicide where they are absent or too few to be of consequence. Although research is being pursued into detection of weed seedlings (e.g. Giselsson *et al*., 2013), most current systems, especially for grass weeds, rely on mapping weeds at maturity to guide spraying decisions in the following crop. Knowing the relationships between weeds and soil could underpin these approaches by identifying where the weeds might persist or spread, based on thresholds of soil variables, for example clay content, in the field. These areas could be sprayed as buffers around existing patches to insure against individuals escaping control. Ultimately, if sufficiently robust models of weed spatial distribution could be developed (incorporating thresholds of soil properties), soil maps could be used as the basis for weed patch spraying decisions. Furthermore, if the coarse‐scale relations observed here are found to be common across additional fields, it is more likely that farmers would adopt variable management at these scales than precision spraying at fine scales.
